# Longitudinal modeling of efficacy response in patients with lupus nephritis receiving belimumab

**DOI:** 10.1007/s10928-024-09907-w

**Published:** 2024-03-29

**Authors:** Monica Simeoni, Shuying Yang, Debra J. Tompson, Richard Dimelow

**Affiliations:** 1 GSK, Clinical Pharmacology Modelling and Simulation, Brentford, Middlesex UK; 2 GSK, Clinical Pharmacology Modelling and Simulation, Gunnels Wood Rd, Stevenage, Hertfordshire SG1 2NY UK

**Keywords:** Efficacy, Exposure response, Monoclonal antibodies, Nephrology, Nonlinear models, Pharmacometrics

## Abstract

Belimumab was approved for active lupus nephritis (LN) in adults in the European Union and patients ≥ 5 years of age in the USA based on a Phase 3, double-blind, placebo-controlled, 104-week study. The study evaluated the efficacy of belimumab plus background standard therapy in adults with active LN using an intravenous (IV) dose of 10 mg/kg. A longitudinal analysis of Primary Efficacy Renal Response (PERR) and Complete Renal Response (CRR) was performed to assess whether patients with high proteinuria at the start of belimumab treatment would benefit from a higher dose. Responder probability was modeled as a logistic regression with probability a function of time and treatment (belimumab or placebo). Dropout risk at each visit was incorporated into a joint model of efficacy response; only efficacy data prior to dropout events (belimumab discontinuation, treatment failure, or withdrawal) were included. Average belimumab concentration over the first 4 and 12 weeks and baseline proteinuria were considered as continuous covariates. In general, renal response (PERR and CRR) over time was higher in patients receiving belimumab than in those receiving placebo. Baseline proteinuria was considered the most relevant predictor of renal response, with reduced efficacy in patients with increased proteinuria for both belimumab or placebo treatment. For belimumab-treated patients, belimumab exposure was not found to be an important predictor of renal response. In conclusion, the 10 mg/kg IV dose was considered appropriate in all patients and there was no evidence to suggest a higher response would be achieved by increasing the dose.

**Supplementary Information:**

The online version contains supplementary material available at 10.1007/s10928-024-09907-w.

## Introduction

Belimumab, a human immunoglobulin G (IgG)1λ monoclonal antibody, is an approved biologic agent for the treatment of active, autoantibody-positive systemic lupus erythematosus (SLE; in patients ≥ 5 years of age in over 60 countries) and active lupus nephritis (LN; in adults in the European Union and patients ≥ 5 years of age in the USA) [1–5]. SLE is a chronic autoimmune disorder, characterized by a broad spectrum of manifestations [6]. LN, a renal manifestation of SLE, occurs in approximately 40% of patients with SLE and is associated with end-stage kidney disease (ESKD) and death [7]. Patients with SLE and LN have been shown to have abnormal B-cell activation and differentiation, as well as elevated serum levels of soluble B-lymphocyte stimulator (BLyS), a key protein involved in the selection and survival of B cells [8–10]. Belimumab is able to bind and inhibit the biological activity of BLyS and has been shown to reduce disease activity, steroid use, and the occurrence of disease flares in several Phase 3 clinical trials [11–14].

The approval of belimumab for treatment of active LN was based on a Phase 3, double-blind, placebo-controlled, 104-week study (BLISS-LN) [15]. The efficacy of intravenous (IV) belimumab in patients with LN was assessed by summarizing the number of patients who achieved a Primary Efficacy Renal Response (PERR) and Complete Renal Response (CRR) [15] and evaluating the probability of experiencing a renal-related event or death [16]. One of the main components assessed in PERR and CRR is proteinuria, which, in patients with LN, results from inflammation-induced glomeruli damage and the excretion of intermediate- and high- molecular-weight proteins (including monoclonal antibodies and IgG) [17]. Belimumab (molecular weight 147 kDa) should not be cleared from the kidneys of patients with normal glomerular permeability due to the size restriction of glomerular filtration (predicted cut-off, 30–50 kDa) [3, 18]. However, glomeruli damage experienced by patients with LN is expected to increase renal elimination and consequently reduce systemic exposure to belimumab [16].

The approved IV dosing regimen for LN (10 mg/kg every 2 weeks for the first three doses, then every 4 weeks thereafter) was based on population pharmacokinetic (PK) modeling and exposure–response analyses using data from the BLISS-LN study [15, 19]. Based on this analysis (**Online Resource 11**), and reported in a US Food and Drug Administration (FDA) review article of these data, it was concluded that there was no adequate information to suggest that a higher dose of belimumab would provide additional benefit for patients with lower exposure due to higher proteinuria [19].

In support of the regulatory submission, we developed a longitudinal model and conducted an analysis of the efficacy response (PERR and CRR) measured over the entire 104-week study period, to investigate whether patients with LN with high proteinuria at the start of belimumab treatment would benefit from a higher dose.

## Methods

### BLISS-LN study

The design and outcomes of the BLISS-LN study (GSK Study 114054; NCT01639339) have been reported previously [15, 16]. In brief, BLISS-LN was a Phase 3, double-blind, placebo-controlled, 104-week study that evaluated the safety and efficacy of belimumab 10 mg/kg IV plus background standard therapy in adults with active LN. The study was conducted across 107 sites; all sites received approval from their respective ethics committees or institutional review boards.

During the BLISS-LN study, blood samples were collected for PK analysis of belimumab serum concentration at the following time points: 0, 3, 14, 28, 56, 168, 171, 364 and 728 days. Urinalysis (proteinuria measured by urine protein/creatinine ratio [uPCR]) and hematology assessments were performed at baseline and every 4 weeks for the duration of the study.

In this longitudinal analysis, the dichotomous PERR and CRR data from BLISS-LN were analyzed. A PERR responder was defined as a patient with an estimated glomerular filtration rate (eGFR) no more than 20% below the pre-flare value or ≥ 60 ml/min/1.73 m^2^ of body surface area, with uPCR ≤ 0.7 (g/g), and who had not received rescue therapy. The more stringent CRR was defined as an eGFR of no more than 10% below the pre-flare value or ≥ 90 ml/min/1.73 m^2^ of body surface area, with uPCR < 0.5 (g/g), and who had not received rescue therapy. If the PERR/CRR responder criteria were not met, patients were classified as non-responders as per a composite variable estimand strategy.

### Longitudinal analysis of efficacy response

A longitudinal logistic model of the responder probability was fitted to individual efficacy response data collected over time from the placebo and active arms of the BLISS-LN study, for each efficacy endpoint (PERR or CRR) separately. Specifically, the model was defined as:

Equation 1: Longitudinal logistic model for PERR or CRR responder rate

$$Logit\left({P}_{RESP}(t)\right)={RR}_{SS} - {\Delta }_{RR}\bullet {e}^{-{{\text{K}}}_{RR}\bullet t}+{\uptheta }_{BEL}\times {\text{TRT}}$$

where: P_RESP_(t) is the probability of response at time t, RR_SS_ defines the responder rate at steady state in placebo, ∆_RR_ defines the overall change in the responder rate from baseline to steady state in placebo, K_RR_ is the rate constant for the change in responder rate over time, and θ_BEL_ is the effect of belimumab therapy on the responder rate. TRT is a binary variable (0 for placebo, 1 for belimumab) to incorporate this belimumab drug effect, which is additional to the placebo responder rate. Inclusion of random effect parameters does not necessarily lead to better parameter estimates for logistic regression models [20], and in our case a random effect could not be identified from the data; therefore, the model did not include random effects.

Model development and covariate selection was conducted using the PERR endpoint, the primary efficacy endpoint for the study used to evaluate the efficacy response achieved for support the 10 mg/kg IV dose in dosing recommendation for all patients with LN. For CRR, the secondary efficacy endpoint, only the final models determined from the PERR endpoint were investigated to explore whether the results were consistent between the two efficacy endpoints.

### Modeling patient dropout

To investigate the impact of patient dropout on model performance, the efficacy response on-treatment was jointly modeled with the risk of dropout [21]. Dropout events included treatment discontinuations, treatment failures, or withdrawal from the study. For this model, the longitudinal efficacy response data (PERR and CRR) up to the last observed response on treatment were included. A constant hazard model was used to model dropout risk where the instantaneous hazard depends on the patient’s responder status at the given time: HZ_R_ for responder and HZ_NR_ for non-responder. The likelihood for an individual with last visit on-treatment at time T1, who subsequently drops out at later time T2 is:

Equation 2: The likelihood function for an individual with PERR or CRR responder rates over time in a joint efficacy and dropout model

$$Likelihood=\left[\prod_{i=1}^{n}{{P}_{RESP}\left({t}_{i}\right)}^{{RR}_{i}}\times {\left(1-{P}_{RESP}\left({t}_{i}\right)\right)}^{1-{RR}_{i}}\right]\times {P}_{SURV}\left(T1\right)\times {P}_{DROP}\left(T2\right)$$

where:

$$\begin{array}{c}{P}_{SURV}\left(T1\right)={e}^{-{\int }_{0}^{T1}HZ(t)\times dt}\\ {P}_{DROP}\left(T2\right)=1-{e}^{-{\int }_{T1}^{T2}HZ(t)\times dt}\end{array}$$

$${{HZ(t) = }}\left\{ \begin{gathered} {{HZ}}_{R} \;\;\;{\text{if}}\;{\text{patient}}\;{\text{is}}\;{\text{a}}\;{\text{responder}}\;{\text{at}}\;{\text{time}}\;{\text{t}} \hfill \\ {HZ}_{{NR}} \;\;{\text{if}}\;{\text{patient}}\;{\text{is}}\;{\text{a}}\;{\text{non - responder}}\;{\text{at}}\;{\text{time}}\;{\text{t}} \hfill \\ \end{gathered} \right.\,\,$$

and RR_1_…RR_n_ is the set of n PERR or CRR observations at corresponding times t_1_…t_n_ ≤ T1 for the individual, where the binary variable RR_i_ represents the observed PERR or CRR at time t_i_ (1 for responder, 0 for non-responder), P_RESP_(t_i_) is the probability of being a responder at time t_i_ (Eq. 1), P_SURV_(T1) is the probability an individual remains on-treatment to time T1 conditional on the observed response through time t, P_DROP_(T2) is the probability the individual subsequently drops out of the study between the last visit on-treatment at time T1 to the dropout event at time T2, and HZ(t) is the hazard pertaining to the instantaneous dropout risk at time t. The observed response was carried forward to construct the hazard over a time interval between observation events. This approach was defined by Hu and Sale as a random dropout model [22] and has also been implemented in the efficacy analysis of mavrilimumab, a treatment for rheumatoid arthritis [23].

### PK analysis

Individual belimumab exposures were derived from a separate population PK analysis of the concentration–time data collected from all belimumab-treated patients in the study. A two-compartmental PK model with first order distribution and elimination was fitted to the PK data. Belimumab clearance was informed by fat-free mass, which described the allometric effects of body size, and the time varying proteinuria and albumin levels, which informed the renal contribution of belimumab clearance in LN (see **Supplementary materials, Online Resource 1** for further details). The individual predicted PK profiles were simulated according to the actual belimumab dose amounts and dose times for each patient; from these profiles the average concentration over the first 4 and 12 weeks of treatment (Cavg4 and Cavg12, respectively) were calculated.

### Covariate selection

No full covariate search was applied. The placebo and active treatment arms of BLISS-LN were randomized over induction therapy (cyclophosphamide or mycophenolate mofetil), and all patients received high-dose corticosteroids. The covariate selection therefore focused only on belimumab exposure or covariates directly linked with exposure, ie, proteinuria. Baseline proteinuria (PROT_BL_) and belimumab exposure (Cavg4 and Cavg12, respectively) were explored by adding them separately into the relevant model parameters and measuring the drop in the objective function value (OBJ). PROT_BL_ assessed the influence of baseline disease severity, and Cavg4 and Cavg12 assessed the influence of early belimumab exposure, on long-term efficacy response. Data from the placebo arm of the study inform the impact of baseline proteinuria on response, whereas data from the belimumab arm inform the additional effects of exposure on response. For nested models, the difference in OBJ was assumed to be approximately χ^2^ distributed, and a significance level of P = 0.001 was used to justify the addition of each parameter requiring an approximate 10-point drop in OBJ.

### Model simulations

Individual patient values for PROT_BL_ and belimumab Cavg4 or Cavg12 were used as the covariate inputs to the model to simulate the patient’s response probability over time. In the joint efficacy and dropout model, the overall response probability was simulated as the product of the response probability on-treatment (P_RESP_(t), Eq. 1) and the survival probability the patient is still on-treatment (P_SURV_(t), Eq. 2). The hazard for the dropout risk was constructed as the sum of the weighted responder and non-responder contributions:

Equation 3: Simulated responder probability over time with weighted hazard

$${P}_{RESP}^{SIM}\left(t\right)={P}_{RESP}\left(t\right)\times {P}_{SURV}\left(t\right)$$

where:

$${P}_{RESP}\left(t\right)={logit}^{-1}({{\text{R}}}_{SS} - {\Delta }_{RR}\bullet {e}^{-{{\text{K}}}_{RR}\bullet t}+{\uptheta }_{BEL}\times {\text{TRT}})$$

$${P}_{SURV}\left(t\right)= {e}^{-{\int }_{0}^{t}HZ(t)\times dt}$$

$$HZ\left(t\right)= H{Z}_{R} \times {P}_{RESP}(t) + H{Z}_{NR} \times (1 - {P}_{RESP}(t))$$

Model simulations sampled the uncertainty in the parameters. Each sampled model was used to simulate the responder probability over time for each patient in the study, based on the patient’s baseline proteinuria and associated belimumab exposure. The median and 95% prediction intervals were calculated and the 95% confidence intervals about the median and prediction intervals were derived by combining the results from each sampled model. The simulated results were compared to the observed responder rates, classifying dropout as being non-responder. The confidence intervals in the observed responder rates were calculated using the exact (Pearson-Clopper) method for a binomially distributed variable.

### Software

All analyses were conducted using NONMEM version 7.3 (ICON Development Solutions, Ellicott City, MD, USA) on a validated GSK modeling platform.

## Results

### Model independent analysis

**Fig. 1:**
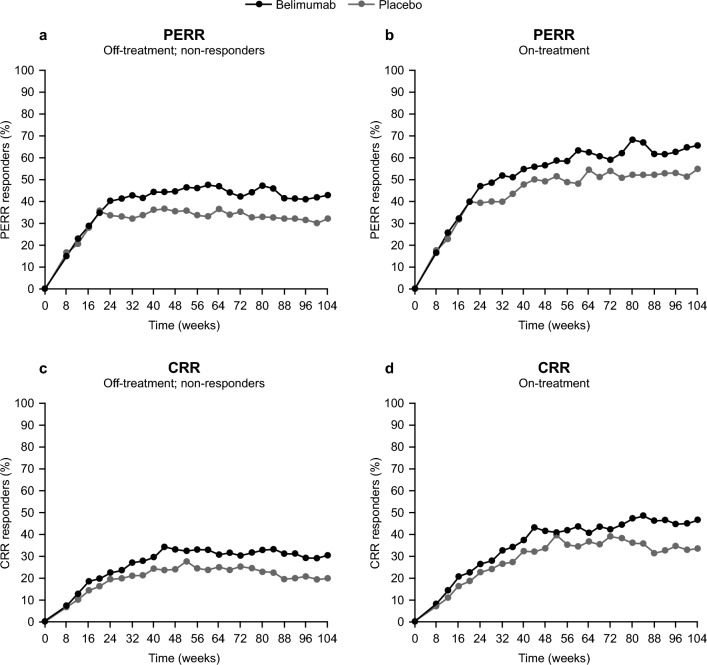
Proportion of PERR responders (**a**, **b**) and CRR responders (**c**, **d**) when imputing off-treatment observations as non-responders (**a**, **c**) and for on-treatment data only (**b**, **d**) by treatment group. *CRR*, Complete Renal Response; *PERR*, Primary Efficacy Renal Response

A total of 448 patients were included in the dataset for the longitudinal modeling, of whom 224 received placebo and 224 received belimumab 10 mg/kg IV (Table 1). The proportions of PERR responders and CRR responders over time were higher in the belimumab group than in the placebo group, with response rates for the more stringent measure of efficacy, the CRR, expectedly lower than for the PERR (Fig. 1). The data also showed a higher risk of patient dropout for non-responders compared with responders (**Online Resource 2**).

**Table 1:** Patient baseline characteristics

Characteristic Median (min, max) unless otherwise stated	Overall (N = 448)	Placebo (N = 224)	Belimumab 10 mg/kg IV (N = 224)
Age (years)	31 (18, 77)	31 (18, 77)	31 (18, 63)
Weight (kg)	60.0 (34.0, 136.9)	60.3 (34.0, 131.2)	59.0 (36.9, 136.9)
Fat-free mass (kg)	38.4 (25.1, 77.4)	38.5 (25.1, 75.5)	38.1 (26.3, 77.4)
Albumin (g/l)	31.0 (14.0, 43.0)	32.0 (14.0, 43.0)	31.0 (16.0, 42.0)
Proteinuria (g/g)	2.50 (0.16, 35.13)	2.47 (0.16, 35.13)	2.61 (0.18, 16.57)
eGFR (ml/min/1.73 m^2^)	99 (14, 243)	98 (22, 243)	99 (14, 208)
BLyS (ng/ml)	0.48 (0.05, 10.51)^a^	0.49 (0.05, 5.40)^b^	0.48 (0.05, 10.51)
Sex, n (%)			
Female	394 (88)	196 (87.5)	198 (88)
Male	54 (12)	28 (12.5)	26 (12)
Race, n (%)			
American Indian	10 (2)	6 (3)	4 (2)
Asian	224 (50)	109 (49)	115 (51)
Black African ancestry	61 (14)	31 (14)	30 (13)
Multiple	5 (1)	3 (1)	2 (1)
White	148 (33)	75 (33)	73 (33)

^a^n = 447; ^b^n = 223

*BLyS* B-lymphocyte stimulator, *eGFR* estimated glomerular filtration rate, *IV* intravenous

### Model development

Model development was based on the PERR versus time dataset. The key models which emerged from the analysis were then applied to the CRR versus time dataset to compare against this more stringent efficacy endpoint. These models are characterized by different combinations of dropout modeling strategies with inclusion of different treatment effect predictors. Specifically, patient dropout was treated in two ways: (1) the PERR and CRR were assigned as non-responder following patient dropout (dropout = non-responder model, a composite variable estimand strategy); (2) the efficacy response on treatment was jointly modeled with the risk of patient dropout (joint efficacy and dropout model, a while-on-treatment estimand strategy). Additionally, exposures as characterized by Cavg4 and Cavg12 were each included as treatment effect covariates to assess the impact of early exposure on renal response. Overall, 12 key models were used in the analysis, as summarized in Table 2. The impact of early exposure on renal response was assessed through model simulations of the joint efficacy-dropout models with Cavg12 as a treatment effect covariate; parameter estimates of these models are summarized in Table 3. Model development proceeded as follows:

**Table 2:** Model summary

Efficacy endpoint	Models	Model type	Objective function value		
			Full dataset (N = 448)	Cavg4 dataset (N = 438)	Cavg12 dataset (N = 423)
PERR	ModelA1	Dropout = non-responder	13,142	12,992	12,617
	ModelA2	+ Cavg4 on θ_BEL_	–	12,948	–
	ModelA3	+ Cavg12 on θ_BEL_	–	–	12,542
	ModelB1	Joint efficacy-dropout	11,476	11,399	11,234
	ModelB2	+ Cavg4 on θ_BEL_	–	11,393	–
	ModelB3	+ Cavg12 on θ_BEL_	–	–	11,213
CRR	ModelC1	Dropout = non-responder	11,021	10,939	10,722
	ModelC2	+ Cavg4 on θ_BEL_	–	10,921	–
	ModelC3	+ Cavg12 on θ_BEL_	–	–	10,690
	ModelD1	Joint efficacy-dropout	10,741	10,669	10,520
	ModelD2	+ Cavg4 on θ_BEL_	–	10,667	–
	ModelD3	+ Cavg12 on θ_BEL_	–	–	10,514

*Cavg4* average concentration between Weeks 0 and 4, *Cavg12* average concentration between Weeks 0 and 12, *CRR* Complete Renal Response, *θ*_*BEL*_ model parameter relating to the belimumab drug effect for PERR or CRR, *PERR* Primary Efficacy Renal Response

**Table 3:** Parameter estimates of selected joint efficacy-dropout models

	PERR endpoint		CRR endpoint	
Estimate (%RSE)^a^	Model B1	Model B3	Model D1	Model D3
RR_SS_ (θ1)	0.161 (± 90.9%)	0.157 (± 93.1%)	− 0.493 (± 31.6%)	− 0.491 (± 31.8%)
∆_RR_ (θ2)	4.61 (± 13.4%)	4.61 (± 13.4%)	5.54 (± 19.8%)	5.54 (± 19.9%)
K_RR_ (θ3) [1/day]	0.0117 (± 14.1%)	0.0117 (± 14.2%)	0.0119 (± 17.0%)	0.0119 (± 17.1%)
θ_BEL_ (θ4)	0.212 (± 83.7%)	0.233 (± 76.5%)	0.0725 (± 275%)	0.106 (± 188%)
PROT_BL_ on RR_SS_ (θ5)	− 0.316 (± 50.0%)	− 0.320 (± 49.2%)	− 0.0684 (± 258%)	− 0.0681 (± 259%)
PROT_BL_ on K_RR_ (θ6)	− 0.00448 (± 22.6%)	− 0.00443 (± 23.4%)	− 0.00575 (± 19.1%)	− 0.00572 (± 19.1%)
PROT_BL_ on θ_BEL_ (θ7)	− 0.577 (± 36.0%)	− 0.509 (± 42.7%)	− 0.676 (± 33.5%)	− 0.650 (± 36.5%)
Cavg12 on θ_BEL_ (θ8)	–	0.500 (± 76.3%)	–	0.186 (± 222%)
Log(HZ_R_)	− 8.46 (± 2.5%)	− 8.46 (± 2.5%)	− 8.62 (± 3.2%)	− 8.62 (± 3.2%)
Log(HZ_NR_/HZ_R_)	1.64 (± 13.9%)	1.49 (± 15.4%)	1.62 (± 18.0%)	1.48 (± 19.7%)

^a^%RSE is calculated as SE/estimate × 100

*Cavg12* average concentration between Weeks 0 and 12, *CRR* Complete Renal Response, *∆*_*RR*_ change in the responder rate over time in placebo, *HZ*_*NR*_ constant hazard used to model dropout risk for non-responders, *HZ*_*R*_ constant hazard used to model dropout risk for responders, *K*_*RR*_ the rate constant of the time course of the responder rate, *PERR* Primary Efficacy Renal Response, *PROT*_*BL*_ baseline proteinuria, *RR*_*SS*_ steady state response, *%RSE* relative SE as percentage of estimate, *SE* standard error, *θ*_*BEL*_ model parameter relating to the belimumab drug effect for PERR or CRR

Initial model development was based on the PERR endpoint with post-dropout response defined as non-responder ModelA1; Table 2). The same model was fitted to the CRR endpoint (ModelC1; Table 2). These models are referred to as the ‘dropout = non-responder models’.

The models for PERR and CRR were then extended to jointly model efficacy on treatment with the risk of dropout as described in the methods (ModelB1 and ModelD1, Table 2). These models are referred to as the ‘joint efficacy-dropout models’. PROT_BL_ was found to be a significant covariate of model parameters RR_SS_, K_RR_ and θ_BEL_ (Eq. 4):

Equation 4: Model parameters of the dropout = non-responder and joint efficacy-dropout models

$$R{R}_{SS} = {\theta }_{1} + {\theta }_{5}\times {\text{log}}\left({PROT}_{BL} / 2.5\right)$$

$${\Delta }_{RR} = {\theta }_{2}$$

$${K}_{RR} = {\theta }_{3} + {\theta }_{6}\times {\text{log}}({PROT}_{BL} / 2.5)$$

$${\theta }_{BEL} = {\theta }_{4} + {\theta }_{7}\times {\text{log}}({PROT}_{BL} / 2.5)$$

where: θ_1_ to θ_4_ are the typical values for each model parameter, θ_5_ to θ_7_ are the covariate parameters which quantify the impact of PROT_BL_ (expressed relative to the baseline median 2.5 g/g) on informing the model parameter values. Consistent with the observational data (**Online Resource 2, Online Resource 6**), the joint efficacy-dropout model results estimated a substantially higher risk of patient dropout for non-responders compared with responders (Log(HZ_NR_/HZ_R_) = 1.64 and 1.62 for ModelB1 and ModelD1, respectively; Table 3).

### Belimumab exposure response models

Belimumab Cavg4 or Cavg12, normalized to their median values (95 μg/ml and 90 μg/ml, respectively), were included as a covariate on the belimumab treatment parameter θ_BEL_ of the dropout = non-responder and joint efficacy-dropout models according to:

Equation 5: Drug treatment model parameter with exposure covariate

$${\theta }_{BEL} = {\theta }_{4} + {\theta }_{7}\times log({PROT}_{BL} / 2.5)+ {\theta }_{8}\times log(Cavg4 / 95)$$

$${\theta }_{BEL} = {\theta }_{4} + {\theta }_{7}\times log({PROT}_{BL} / 2.5) + {\theta }_{8}\times log(Cavg12 / 90)$$

where: θ_8_ is the covariate parameter quantifying the impact of exposure on informing the belimumab treatment effect. The model parameterization links belimumab exposure with renal response for the 10 mg/kg IV dose, as administered in the BLISS-LN study [15]. In this regard, the model is suitable to investigate whether variability in exposure at this specific dose explains any component of variability in renal response. Due to the narrow range of exposures studied (Cavg12 95% prediction interval of 41–159 µg/ml) the shape of the exposure–response relationship could not be identified outside these exposure limits and the model does not necessarily extrapolate to other dose levels. Each exposure–response model was refitted to the PERR and CRR versus time datasets (ModelA2/A3 and ModelB2/B3 for PERR; ModelC2/C3 and ModelD2/D3 for CRR; Table 2).

### Impact of exposure on the statistical fit to the data

Estimates of Cavg4 and Cavg12 were not available for those belimumab-treated patients who dropped out prior to Weeks 4 and 12, respectively. Therefore, the exposure–response models with Cavg4 and Cavg12 as the treatment effect covariate were fitted to reduced datasets containing all placebo-treated patients and only those belimumab-treated patients on treatment at Week 4 or Week 12, respectively: Cavg4 Dataset (n = 438) and Cavg12 Dataset (n = 423) versus the overall dataset (n = 448). The drop in the OBJ was evaluated with respect to the parent model without the exposure covariate. However, for this assessment the parent model OBJ was recalculated by only summing over the individual objective function values for patients in the reduced dataset such that the OBJ calculated for both the parent and the exposure–response models were based on the same group of patients (Table 2).

**Fig. 2:**
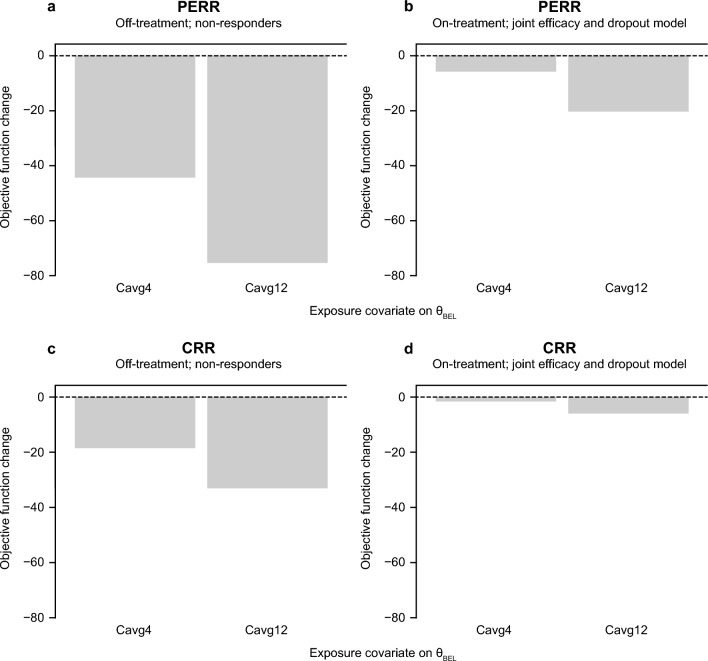
Objective function change when including belimumab Cavg4 or Cavg12 as a covariate on θ_BEL_ for PERR (**a**, **b**) and CRR (**c**, **d**) when imputing off-treatment observations as non-responders (**a**, **c**) and when on-treatment efficacy was jointly modeled with dropout risk (**b**, **d**). *Cavg4*, average concentration between Weeks 0 and 4, *Cavg12*, average concentration between Weeks 0 and 12; *CRR*, Complete Renal Response; *θ*_*BEL*_, model parameter relating to the belimumab drug effect for PERR or CRR; *PERR*, Primary Efficacy Renal Response

Belimumab exposure was found to be a significant covariate of renal response when off-treatment observations were imputed as non-responder; however, the impact of exposure was much smaller (although still statistically significant for Cavg12) when on-treatment efficacy was jointly modeled with dropout risk (Fig. 2). The joint efficacy-dropout models with belimumab Cavg12 as the treatment effect covariate (the models with the lowest OBJ) were selected to evaluate the impact of proteinuria and exposure variability on efficacy in the patient population (ModelB3 for PERR and ModelD3 for CRR; Table 1).

### Proteinuria versus exposure as the driver of clinical response

**Fig. 3:**
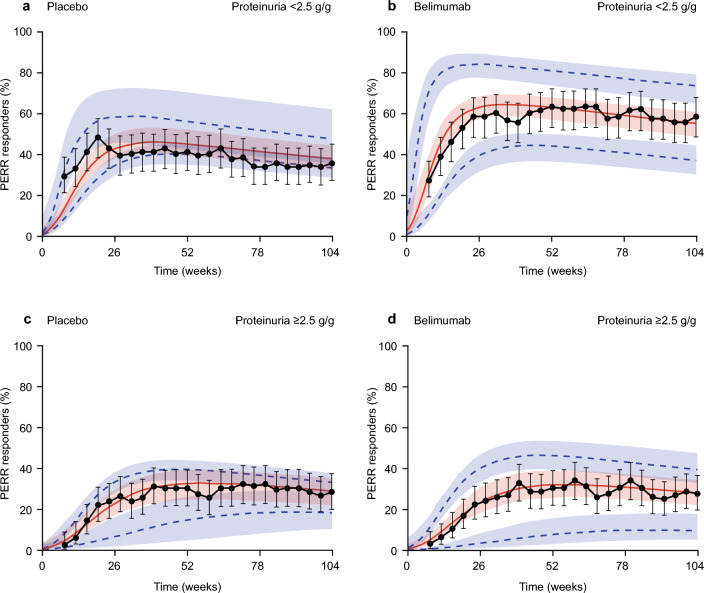
PERR response probability versus time in patients receiving placebo (**a**, **c**) and belimumab (**b**, **d**), observed and simulated from the model, stratified by baseline proteinuria (< 2.5 g/g [a, b] or ≥ 2.5 g/g [**c**, **d**]). Observed response of study BLISS-LN (black points) and 95% CIs (black error bars). The model predicted population median response (red solid line), and 95% prediction intervals (blue dotted lines) with 95% CIs (shaded areas). *CI* confidence interval, *PERR* Primary Efficacy Renal Response

**Fig. 4:**
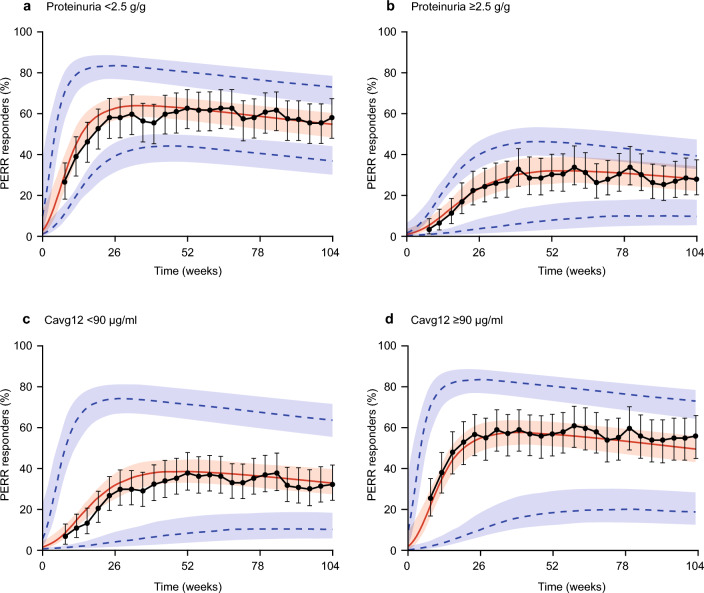
PERR response probability versus time in patients receiving belimumab, observed and simulated from the model, stratified by baseline proteinuria (**a**, **b**) and Cavg12 (**c**, **d**). Observed response of study BLISS-LN (black points) and 95% CIs (black error bars). The model predicted population median response (red solid line), and 95% prediction intervals (blue dotted lines) with 95% CIs (shaded areas). *Cavg12* average concentration between Weeks 0 and 12, *CI* confidence interval, *CRR* Complete Renal Response, *PERR* Primary Efficacy Renal Response

Model simulations showed that the PERR and CRR rates are sensitive to PROT_BL_ levels, with generally lower responder rates and reduced belimumab treatment effect relative to placebo in patients with PROT_BL_ ≥ 2.5 g/g, the median PROT_BL_ in the study (Fig. 3 for PERR and **Online Resource 3** for CRR). For patients receiving belimumab, the observed data also indicate that the response is slightly higher in patients with high (Cavg12 ≥ 90 μg/ml) versus low (Cavg12 < 90 μg/ml) exposure, as confirmed by the simulated median response (Fig. 4 for PERR and **Online Resource 4** for CRR); however, when between-patient variability is considered, as given by the model-derived 95% prediction interval in the responder probability, the low- and high-exposure subgroups are comparable and the impact of exposure on response is small relative to the much larger impact of PROT_BL_ (Fig. 4 for PERR and **Online Resource 4** for CRR).

**Fig. 5:**
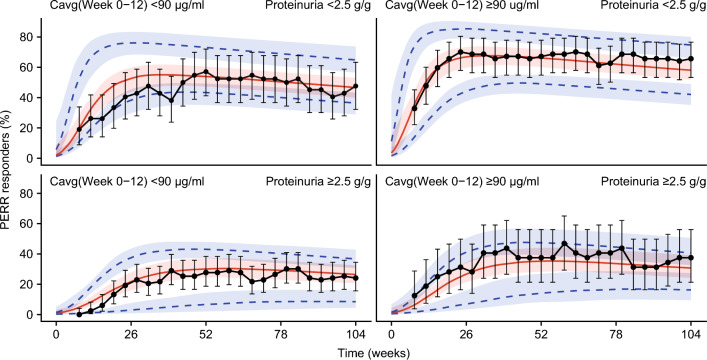
PERR response probability versus time in patients receiving belimumab, observed and simulated from the model, stratified by baseline proteinuria and Cavg12 simultaneously. Observed response of study BLISS-LN (black points) and 95% CIs (black error bars). The model predicted population median response (red solid line), and 95% prediction intervals (blue dotted lines) with 95% CIs (shaded areas). *Cavg12* average concentration between Weeks 0 and 12, *CI* confidence interval, *CRR* Complete Renal Response, *PERR* Primary Efficacy Renal Response

In addition, stratifying the model simulations simultaneously by PROT_BL_ and Cavg12 further supports the result that renal response is not sensitive to early exposure at the 10 mg/kg IV dose in patients with relatively low baseline proteinuria (< 2.5 g/g), but specifically also in patients with high baseline proteinuria (≥ 2.5 g/g) (Fig. 5 and **Online Resource 5**).

## Discussion

### Model development summary

The current analysis describes the longitudinal modeling of the efficacy response of belimumab in patients with LN from the BLISS-LN study on the efficacy endpoints PERR and CRR, which are composite endpoints of the eGFR and proteinuria response. The analysis modeled the binary (responder vs non-responder) PERR and CRR endpoints directly as a logistic regression, rather than modeling the eGFR and proteinuria over time to subsequently derive the efficacy response. The reason for choosing this approach was for simplicity, to fit to a single efficacy endpoint, whereas fitting to two correlated components of the endpoint may have resulted in a more complex model, greater residual variability and loss of model accuracy. Furthermore, in general, not all binary endpoints are derived from continuous components, or not all subcomponents of a composite endpoint may be available for analysis. This work demonstrates an all-purpose modeling approach that can be applied in these situations.

Model development and selection was based on the PERR efficacy endpoint, and the CRR analysis was based on the key models developed for the PERR analysis, such that the same comprehensive selection procedure performed for the PERR was not repeated. This approach was considered appropriate to explore whether the impact of PROT_BL_, the treatment effect of receiving belimumab over placebo, and belimumab exposure in belimumab-treated patients were consistent across these two endpoints. The advantage of using a longitudinal logistic model is that all observed individual data over the 104-week study period are integrated to derive conclusions, and not just the Week 104 endpoint data at the end of the study.

Initial model development was based on the dataset where the renal response (PERR or CRR) following patient dropout—due to investigational product discontinuation, treatment failure, or withdrawal—was imputed as non-responder. While patient dropout during large Phase 3 trials is not unusual, a joint efficacy and dropout model ensures the treatment effect over time is separated from the many diverse factors that can affect patient dropout, and therefore the true treatment effect of the drug may be more reliably inferred from the data. For this reason, further analysis was conducted using a joint efficacy and dropout model fitted to the PERR and CRR versus time data, which deconvolutes the risk of dropout from the on-treatment efficacy response, such that the impact of imputing off-treatment response to non-responders could be evaluated.

Characterization of exposure–response relationships for monoclonal antibodies can often be confounded by various factors that influence both exposure and response, thereby impacting the ability to derive the true, causative exposure–response relationship from the data [24]; collecting data from multiple dose levels to deconvolute confounding covariate effects is important. The current study (BLISS-LN) is restricted to just placebo and 10 mg/kg IV treatment arms, and for the purpose of this modeling, the key covariates of interest were PROT_BL_, treatment effect (belimumab or placebo treatment) and the average belimumab concentration over the first 4 and 12 weeks of treatment in belimumab-treated patients. The placebo arm informs the impact of proteinuria on response and the belimumab arm informs the additional belimumab treatment effect; nevertheless, the exposure–response assessment in belimumab-treated patients is confounded by an association between proteinuria and belimumab exposure, and between proteinuria and the PERR and CRR efficacy endpoints (both of which have proteinuria as a component in their definition of response). A similar type of confounding effect was noted in a US FDA publication that evaluated the exposure response for the cancer treatment nivolumab, in which clearance of nivolumab was time-dependent and associated with improved disease status [25]. Based on the recommendations made by Liu et al. [25], the exposure metrics for the covariate analysis in the current study were based on belimumab exposure early in the study. The average belimumab concentration over the first 4 weeks of treatment was selected to represent the initial exposure following the first dose of belimumab and the loading dose at Week 2 while the average belimumab concentration over the first 12 weeks of treatment was selected to represent the early time period over which the average belimumab exposure was lowest, due to relatively high levels of proteinuria at the start of treatment and the time elapsed since the Week 2 loading dose. The average belimumab exposures over the first 4 and 12 weeks were not imputed for patients who dropped out prior to Weeks 4 or 12, respectively, so the datasets used to evaluate the exposure–response relationship contained slightly fewer patients than the overall dataset. An alternative approach to potentially overcome this limitation would be to develop a more mechanistic-based model integrating all available PK and efficacy data, where the full PK profile is used to drive the efficacy response over time. Future investigation could test the utility of this approach.

### Model conclusions: effects of proteinuria

The effect of PROT_BL_ was considered the most relevant predictor of clinical response (PERR and CRR), with reduced response and reduced treatment effect for belimumab for increased proteinuria at baseline. The results supported a significant separation between the efficacy response rates in patients treated with placebo and belimumab with PROT_BL_ levels < 2.5 g/g, but this treatment difference on the renal response endpoints was less apparent when PROT_BL_ was ≥ 2.5 g/g (Fig. 3). Reasons for this reduced treatment difference on the renal response endpoints for high PROT_BL_ are unclear, but could be a result of irreversible loss of renal function, high localized renal inflammation, which cannot be targeted by circulating belimumab, or that proteinuria reduction is a long-term process and a potential treatment difference is not shown within the 104-week study period [19, 26].

### Model conclusions: effect of exposure (belimumab subgroup analysis)

For belimumab-treated patients, in terms of the effect of belimumab exposure on the PERR and CRR, the results obtained imputing off-treatment observations as non-responder differed from the joint model of on-treatment efficacy and risk of dropout. Specifically, the joint efficacy–dropout model showed that belimumab exposure over the first few weeks of treatment is not a strong predictor of PERR or CRR in belimumab-treated patients (Fig. 4 and Fig. 5). For patients with PROT_BL_ < 2.5 g/g, the treatment difference favoring belimumab over placebo is not sensitive to exposure and therefore is expected to be optimal for the 10 mg/kg IV dose. Likewise, the absence of a treatment difference for patients with PROT_BL_ ≥ 2.5 g/g is perhaps due to high disease activity limiting the potential benefits of belimumab on this renal response endpoint and is not due to reduced exposure in these patients with high proteinuria; specifically, a larger treatment difference over placebo is not expected by increasing the dose. These simulation results are consistent with belimumab’s mechanism of action, where exposure following 10 mg/kg IV is sufficient for saturable binding and complete neutralization of BLyS activity in circulation.

In contrast, when imputing off-treatment observations as non-responders, inclusion of early belimumab exposure as a covariate was highly statistically significant. This result further highlights the importance of selecting an appropriate modeling approach, which can correct for the bias introduced by imputation methods, such as by using Informative dropout models [21]. In fact, when characterizing the exposure–response over a narrow exposure range from single dose level study data, separating drug-related efficacy from patient dropout ensures that the treatment effect over time is correctly inferred from a longitudinal analysis.

### Model conclusions: advantages of a model-based approach

Approval of the 10 mg/kg IV dose for active LN was based, in part, on comparing early belimumab exposure with the PERR and CRR observed at Week 104 at the end of the study (**Online Resource 11**). After accounting for baseline proteinuria, the absence of a strong exposure–response relationship implied belimumab exposures were near the maximal phase of the exposure–response relationship, which supported the recommendation that 10 mg/kg IV was appropriate to treat all patients, including those patients with high proteinuria and therefore relatively low exposure. The model-based longitudinal analysis presented here was an additional exercise to assess the exposure–response relationship; the results were consistent with those from the standard analysis supporting approval of the 10 mg/kg IV dose. The main advantage of a model-based approach is that the exposure–response relationship is derived using all efficacy data collected over the 104-week study period, rather than restricted to just the observed efficacy at a single time point (e.g. end of study at Week 104 only). Therefore, the exposure–response relationship derived from the model may be more robust and less sensitive to outlier data points, increasing confidence when inferring the response expected for other dose levels. Of note, a model-based approach requires sufficient data to enable all model parameters to be identified and estimated with reasonable precision. The BLISS-LN study only included placebo and 10 mg/kg IV arms, and the narrow belimumab exposure range from the 10 mg/kg IV arm was not sufficient to enable a fully integrated PK-PD model from being developed. However, a longitudinal model of the PERR endpoint was developed from the study data and the influence of exposure on the renal response was assessed by integrating a measure of early exposure (Cavg12) as a model covariate.

## Conclusion

In this analysis, longitudinal logistic modeling of efficacy, accounting for the risk of dropout, showed that the principal determinants of response were baseline proteinuria and whether a patient received belimumab 10 mg/kg IV; however, efficacy in belimumab-treated patients was not sensitive to belimumab exposure at this dose. Thus, based on the analysis of these data from a single dose level, there was no evidence to suggest that a higher response would be achieved by increasing the dose, and so the 10 mg/kg IV belimumab dose was considered appropriate to treat all patients.

### Supplementary Information

Below is the link to the electronic supplementary material.

Supplementary file4 (DOCX 1480 KB)

## Data Availability

Anonymized individual patient data and study documents can be requested for further research from www.clinicalstudydatarequest.com.
