# Supplementary material for: Longitudinal modeling of efficacy response in patients with lupus nephritis receiving belimumab
Source: J Pharmacokinet Pharmacodyn. 2024 Mar 29;51(3):289–301. doi: 10.1007/s10928-024-09907-w (PMC11136851; doi:10.1007/s10928-024-09907-w)
Supplement: Supplementary file 1 — Supplementary file4 (DOCX 1480 KB) [file 10928_2024_9907_MOESM1_ESM.docx]

# Supplementary Materials

**Longitudinal modeling of efficacy response in patients with lupus nephritis receiving belimumab**

*Journal of Pharmacokinetics and Pharmacodynamics*

Monica Simeoni, Shuying Yang, Debra J. Tompson, Richard Dimelow

Address correspondence to:

**Richard Dimelow, PhD**

Email: Richard.x.Dimelow@gsk.com

Online Resource 1. Methods

**Pharmacokinetics model developed for patients with LN**

Belimumab pharmacokinetics (PK) in adult and pediatric patients with systemic lupus erythematosus (SLE) is well established [1, 2]. The PK follows two compartmental kinetics with first order distribution and elimination. Body weight (adults) and fat-free mass (children) captures the allometric effects of body weight on belimumab PK. Albumin was also found to be a strong predictor of belimumab clearance in adult patients with SLE (but surprisingly not in children). This may be explained because albumin informs the degree of neonatal Fc receptor (FcRn)‐mediated recycling, protecting immunoglobulin G (IgG) monoclonal antibodies (also albumin and endogenous IgG) from degradation.

A population analysis on the PK data collected in the BLISS-LN study was performed to characterize belimumab PK in adults with lupus nephritis (LN). For patients with LN, fat-free mass was found to best inform the allometric effects of body size on belimumab PK. Increased renal inflammation in LN is associated with proteinuria and expected renal elimination of belimumab [3]. Over time, in response to treatment, proteinuria is reduced, leading to normalization of albumin levels and a reduction in belimumab renal clearance [3]. The time-dependent nature of belimumab clearance in adults with active LN, due to reduction in proteinuria, was characterized in a population PK model (**Online Resource 7**). Goodness of fit plots for the model are shown in **Online Resource 8 and 9.**

In general, higher belimumab exposure correlates with lower proteinuria, either because high exposure achieves a greater treatment effect, resulting in greater proteinuria reduction, or because lower proteinuria means less drug is renally excreted and so exposures are higher (**Online Resource 10**).

In contrast, there was no apparent correlation between efficacy (PERR or CRR after 104 weeks treatment) and early belimumab exposure providing the analysis was stratified by baseline proteinuria (**Online Resource 11**). The absence of an exposure–response relationship implied that exposure following the 10 mg/kg IV dose was sufficiently high to achieve maximum response in all patients. This key exposure–response output, based on the observed renal response, was the foundation for recommending that 10 mg/kg IV was sufficient in all patients, including those patients with high baseline proteinuria and therefore lower initial belimumab exposure.

The aim of the model-based analysis described in this manuscript was to evaluate the effect of belimumab exposure on efficacy response using all efficacy data collected across the 104-week clinical study, to see whether the data support the conclusion that the 10 mg/kg IV dose was sufficient in all patients.

1. Dimelow R, Ji B, Struemper H (2021) Pharmacokinetics of Belimumab in Children With Systemic Lupus Erythematosus. Clin Pharmacol Drug Dev 10 (6):622–633. <https://doi.org/10.1002/cpdd.889>

2. Struemper H, Thapar M, Roth D (2018) Population Pharmacokinetic and Pharmacodynamic Analysis of Belimumab Administered Subcutaneously in Healthy Volunteers and Patients with Systemic Lupus Erythematosus. Clin Pharmacokinet 57 (6):717–728. <https://doi.org/10.1007/s40262-017-0586-5>

3. European Medicines Agency (2021) Benlysta (belimumab): Assessment report. <https://www.ema.europa.eu/en/documents/variation-report/benlysta-h-c-2015-ii-0080-epar-assessment-report-variation_en.pdf>. Accessed 10 February 2022

**Online Resource 2. Proportion of patients who discontinued from study treatment over time, according to their last PERR (a, b) or CRR (c, d) observation prior to discontinuation for non-responders (a, c) and responders (b, d) by treatment group**


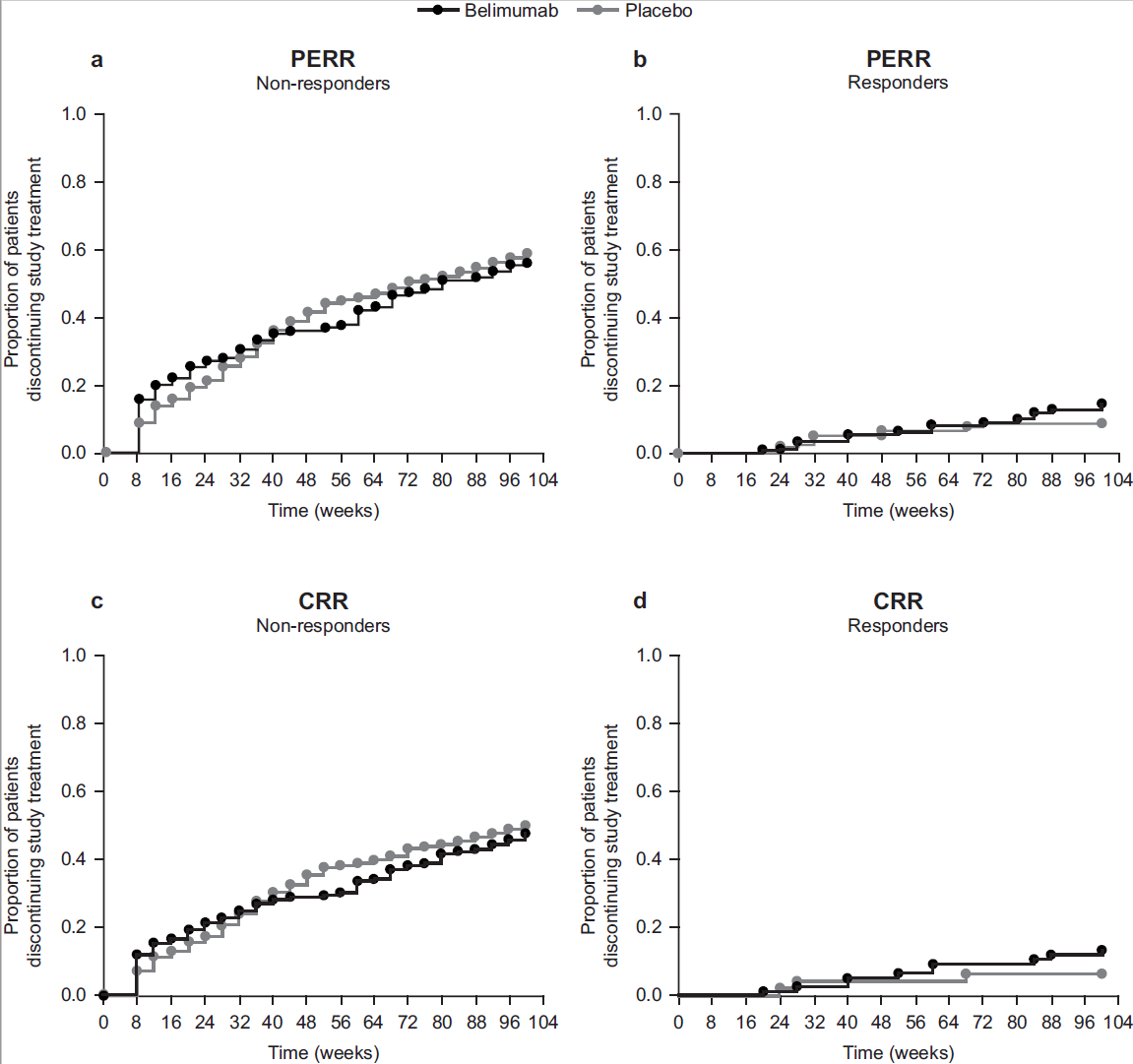


CRR, Complete Renal Response; PERR, Primary Efficacy Renal Response.

Online Resource 3. CRR response probability versus time in patients receiving placebo (a, c) and belimumab (b, d), observed and simulated from the model, stratified by baseline proteinuria (<2.5 g/g [a, b] or ≥2.5 g/g [c, d])


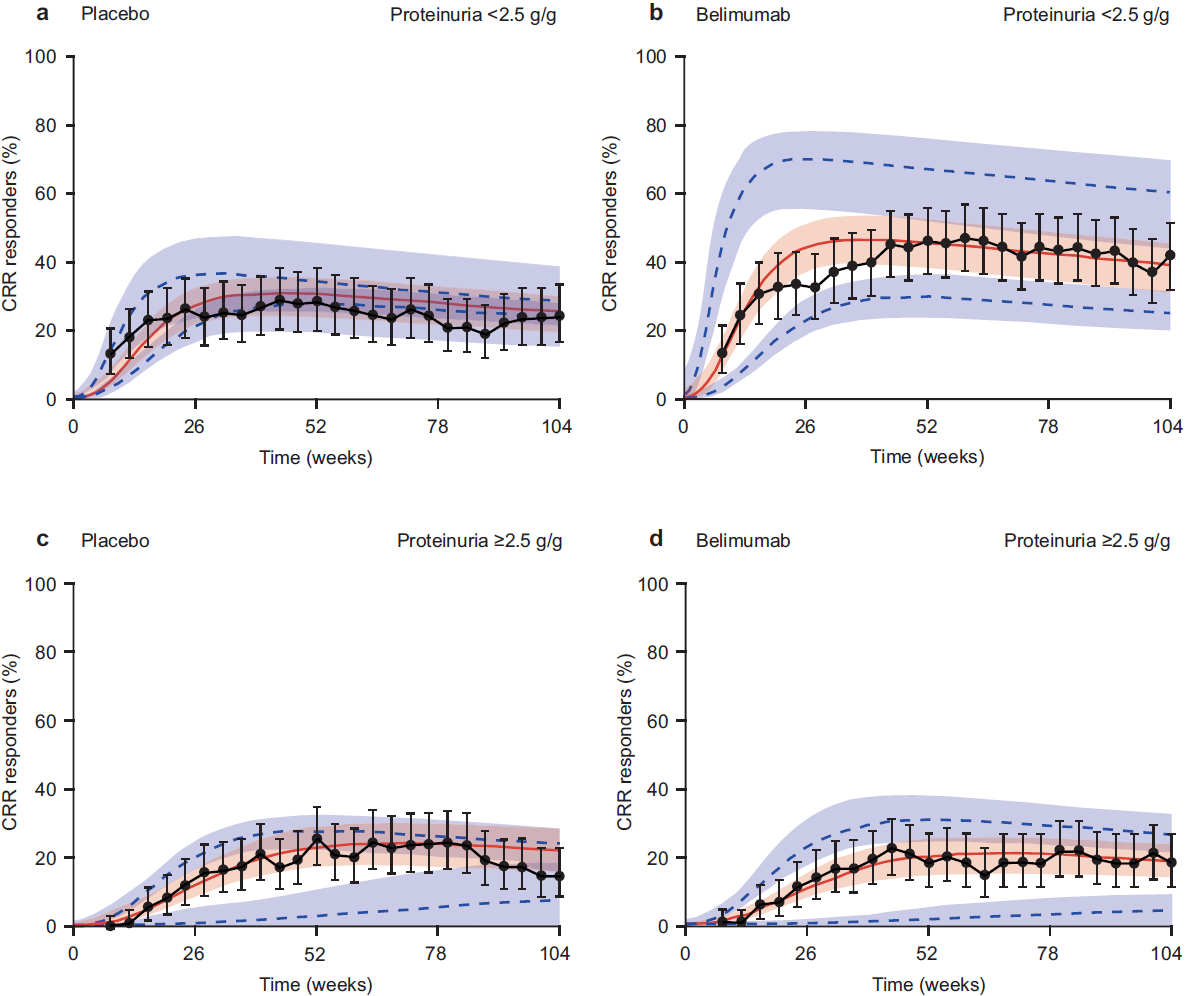


Observed response of study BLISS-LN (black points) and 95% CIs (black error bars). The model predicted population median response (red solid line), and 95% prediction intervals (blue dotted lines) with 95% CIs (shaded areas).

CI, confidence interval; CRR, Complete Renal Response.

Online Resource 4. CRR response probability versus time in patients receiving belimumab, observed and simulated from the model, stratified by baseline proteinuria (a, b) and Cavg12 (c, d)


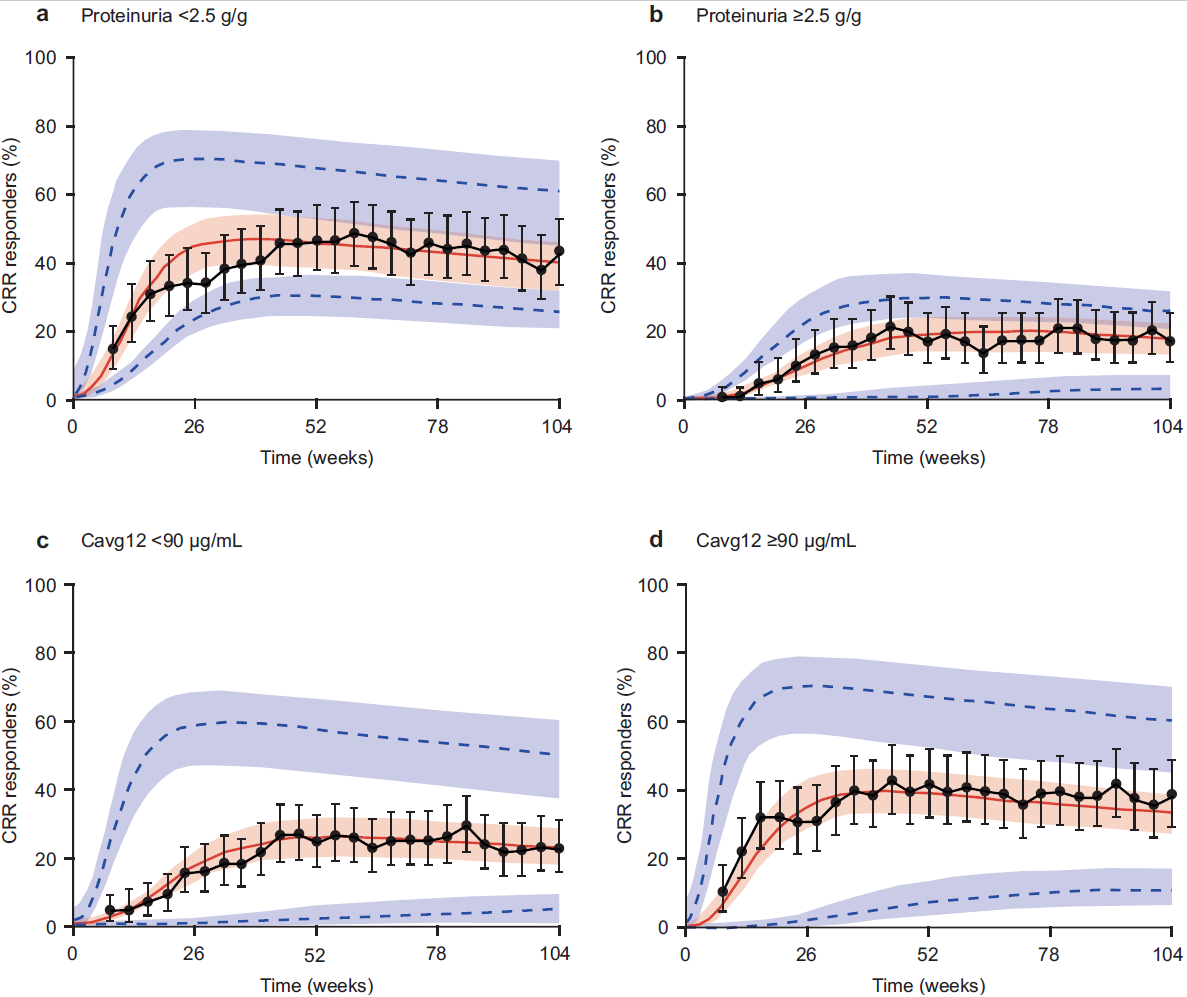


Observed response of study BLISS-LN (black points) and 95% CIs (black error bars). The model predicted population median response (red solid line), and 95% prediction intervals (blue dotted lines) with 95% CIs (shaded areas).

Cavg12, average concentration between Weeks 0 and 12; CI, confidence interval; CRR, Complete Renal Response.

Online Resource 5. CRR response probability versus time in patients receiving belimumab, observed and simulated from the model, stratified by baseline proteinuria and Cavg12 simultaneously


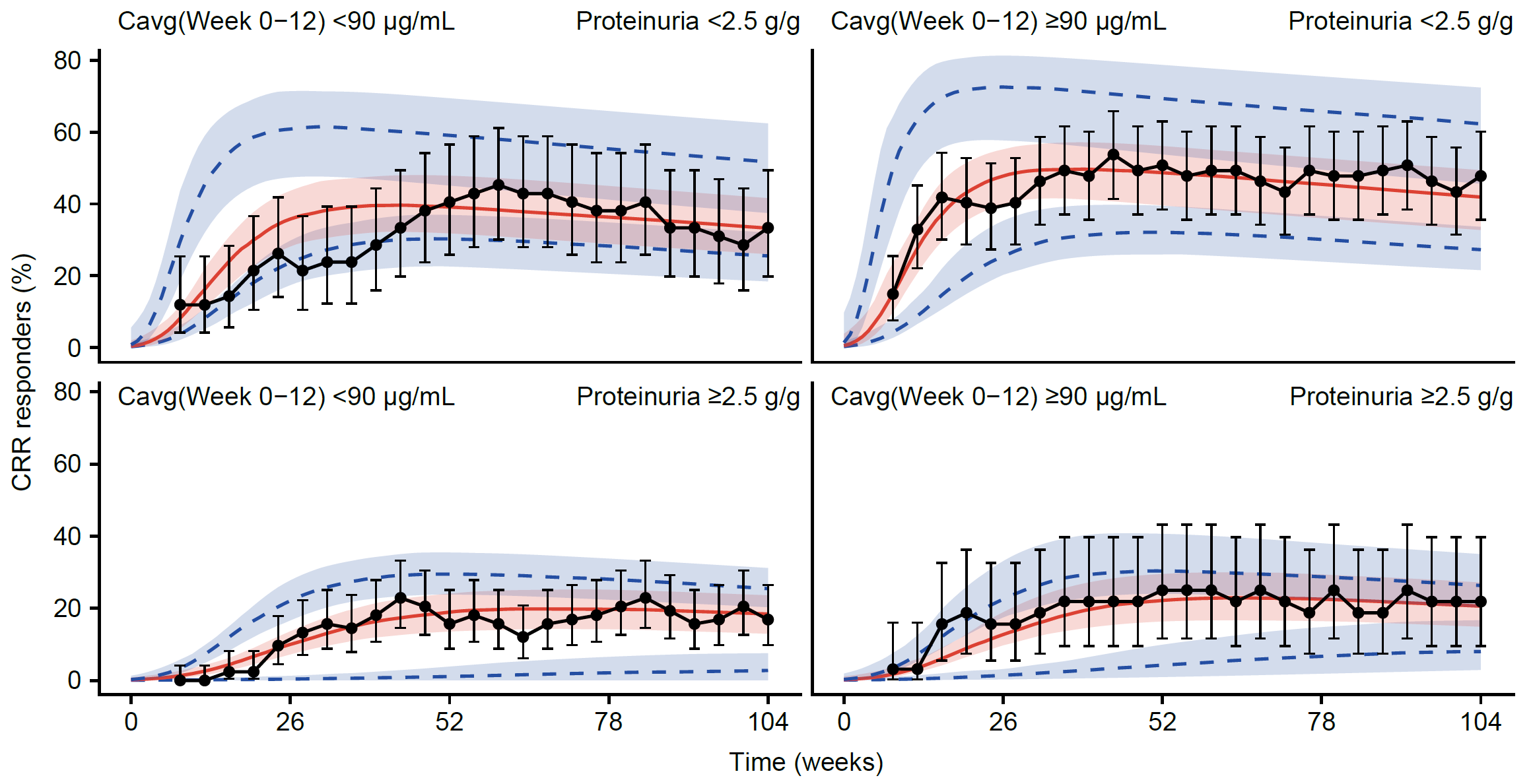


Observed response of study BLISS-LN (black points) and 95% CIs (black error bars). The model predicted population median response (red solid line), and 95% prediction intervals (blue dotted lines) with 95% CIs (shaded areas).

Cavg12, average concentration between Weeks 0 and 12; CI, confidence interval; CRR, Complete Renal Response.

Online Resource 6. Proportion of patients who dropped out over time derived using the joint efficacy-dropout model for (a) PERR and (b) CRR


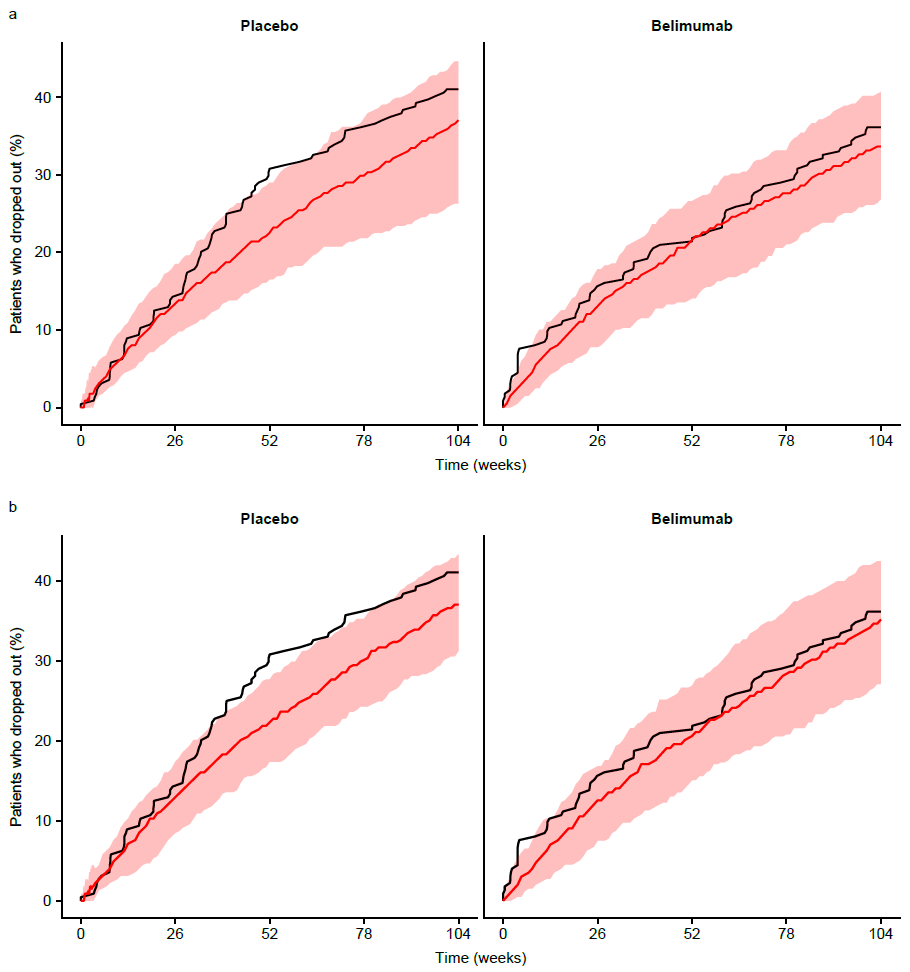


The observed dropout probability in study BLISS-LN (black solid line) is superimposed on the simulated dropout probability median (red solid line) and associated 95% confidence interval (light red area).

Online Resource 7. Fixed and random effect parameters of the PK model

| **Fixed Effect Parameters** | **Model Point Estimate (%RSE)** | **Model Point Estimate 95% CI** |
| --- | --- | --- |
| CL (ml/day) | 175 (2.7) | 165, 184 |
| x (FFMBL/38.4)^θ^ | 0.624 (16.5) | 0.422, 0.825 |
| x (ALB / 42)^θ1/(1+ θ2×PROT)^ |  |  |
| θ1 | –1.74 (6.1) | –1.95, –1.53 |
| θ2 | 0.0832 (39.5) | 0.0187, 0.148 |
| x (1 + θ×PROT) | 0.0663 (24.6) | 0.0344, 0.0983 |
| V1 (ml) | 2728 (1.8) | 2630, 2825 |
| x (FFMBL/38.4)^θ^ | 0.723 (13.5) | 0.531, 0.914 |
| Q (ml/day) | 487 (6.3) | 427, 547 |
| x (FFMBL/38.4)^θ^ | See FFMBL on CL | - |
| V2 (ml) | 1992 (4.99) | 1797, 2187 |
| x (FFMBL/38.4)^θ^ | See FFMBL on V1 | - |
| **Residual variability (Normally Distributed)** | | |
| PROP | 0.241 (4.5) | - |
| ADD (µg/ml) | 0.1 (Fixed) | - |
| **Inter-individual variability**  **(Log-Normally Distributed)** | **Model Point Estimate (%RSE)** | **Model Point Estimate %CV** |
| *w^2^_CL_* | 0.0593 (16.7) | 24.7 |
| *w^2^_V1_* | 0.0322 (33.7) | 18.1 |
| *w^2^_V2_* | 0.133 (34.3) | 37.7 |
| *w^2^_PROP_* | 0.346 (14.9) | 64.3 |

%RSE, relative standard error as percentage of estimate; ADD, additive residual parameter; ALB, albumin level (g/L); CI, confidence interval; CL, clearance; CV%, the coefficient of variation calculated as √(exp(ω2) – 1); FFMBL, fat-free mass at baseline (kg); PROP, proportional residual parameter; PROT, proteinuria level (g/g); Q, inter-compartmental flow rate; θ, albumin covariate parameter; V1, volume of distribution for the central compartment; V2, volume of distribution for the peripheral compartment; *ω^2^_CL_*, between-patient log-scale variance on CL; *ω^2^_V1_*, between-patient log-scale variance on V1; *ω^2^_V2_*, between-patient log-scale variance on V2; *ω^2^_PROP_*, between-patient log-scale variance on PROP.

Online Resource 8. Goodness of fit plots for the PK model


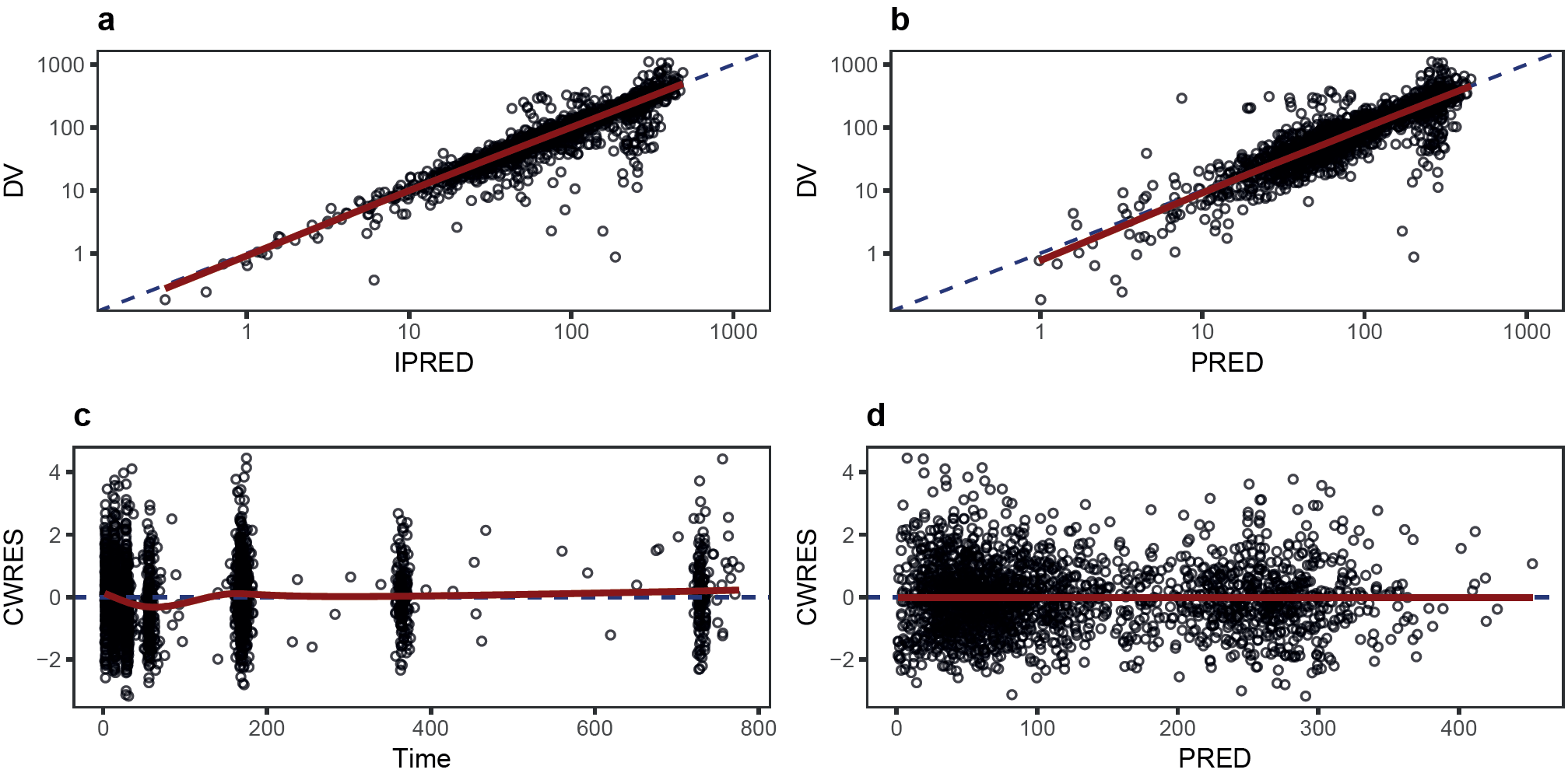


CWRES, conditional weight residual; DV, dependent variable; IPRED, individual predicted belimumab concentrations; PRED, population median predicted belimumab concentration.

Online Resource 9. Visual predictive check for the PK model


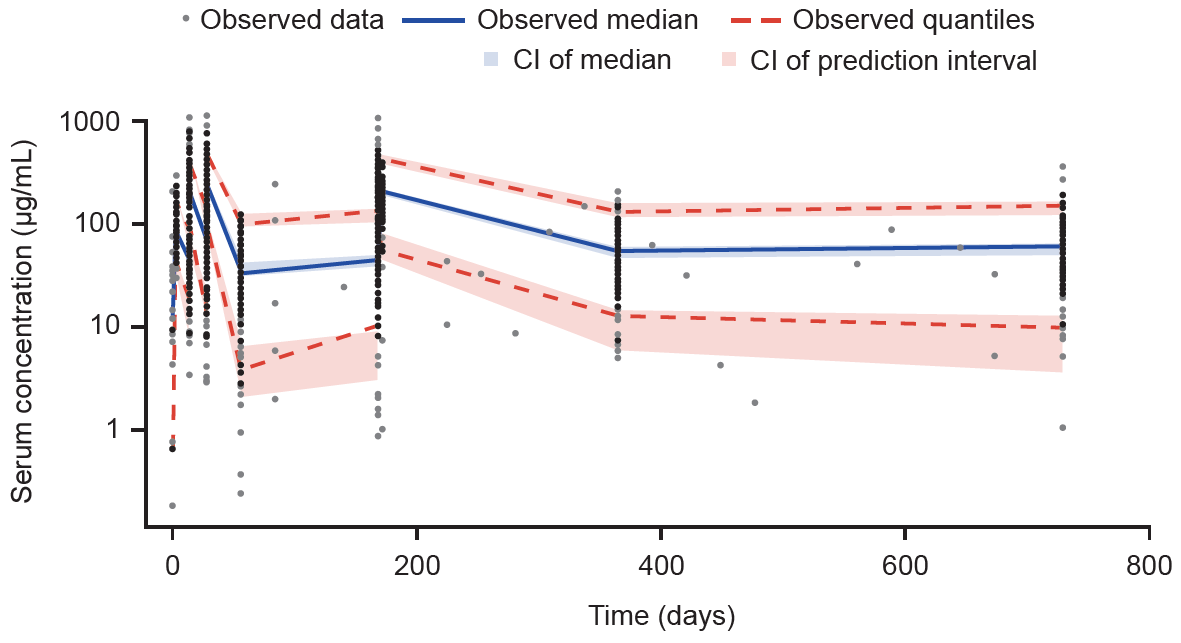


Belimumab concentration versus time after first dose. Observed data (black points), observed 2.5th and 97.5th percentiles (dotted red line) with simulated 95% CI (shaded red regions); observed 50th percentile (solid blue line) with simulated 95% CI (shaded blue region).

CI, confidence interval.

Online Resource 10. Proteinuria versus belimumab exposure derived using the PK model


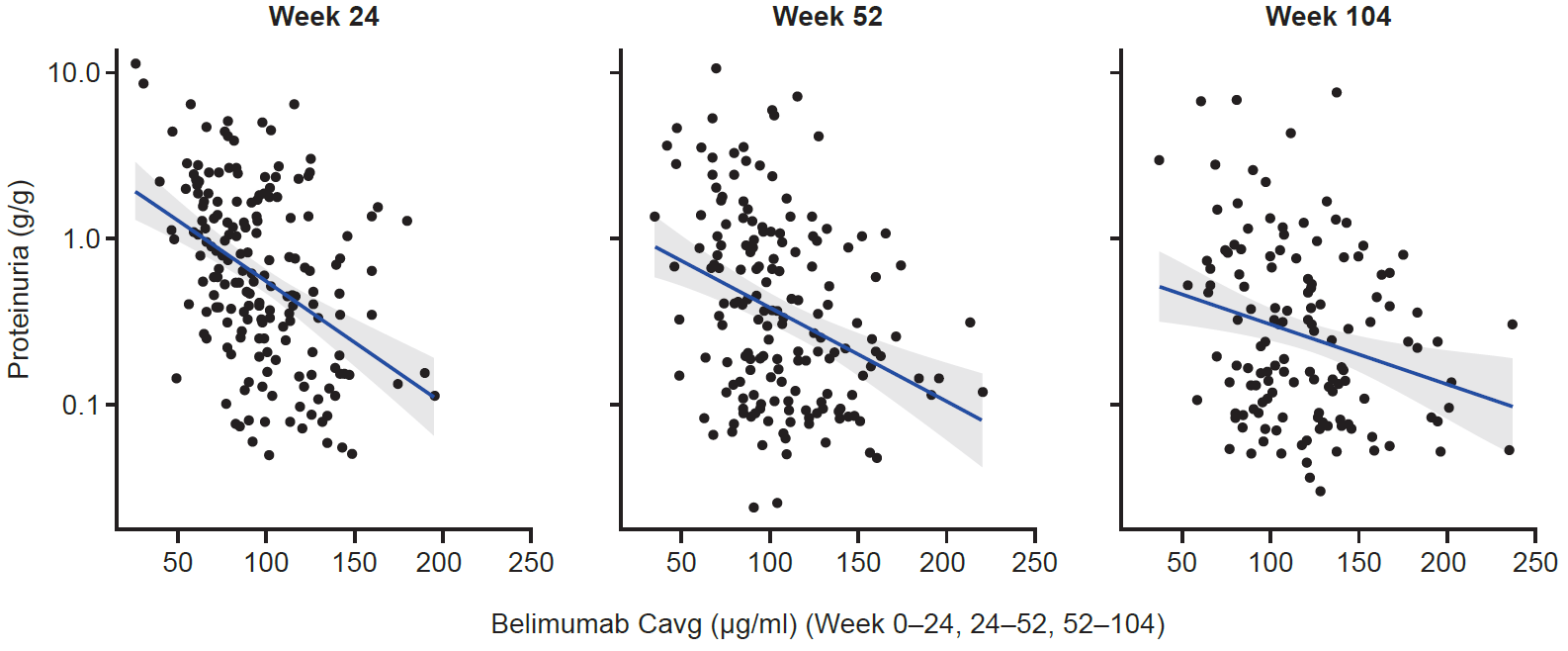


Cavg is calculated from Weeks 0 to 24 (left panel), Weeks 24 to 52 (central panel), and Weeks 52 to 104 (right panel). Individual data points (black points) with linear regression line (solid blue line) and 95% confidence interval (grey shaded region). Under the null hypothesis of a zero gradient, p-values are <0.001 (left panel), <0.001 (center panel), and 0.003 (right panel).

Cavg, average concentration.

Online Resource 11. Belimumab exposure distribution for (a) PERR and (b) CRR responder and non-responder subgroups stratified by baseline proteinuria above and below 2.5 g/g


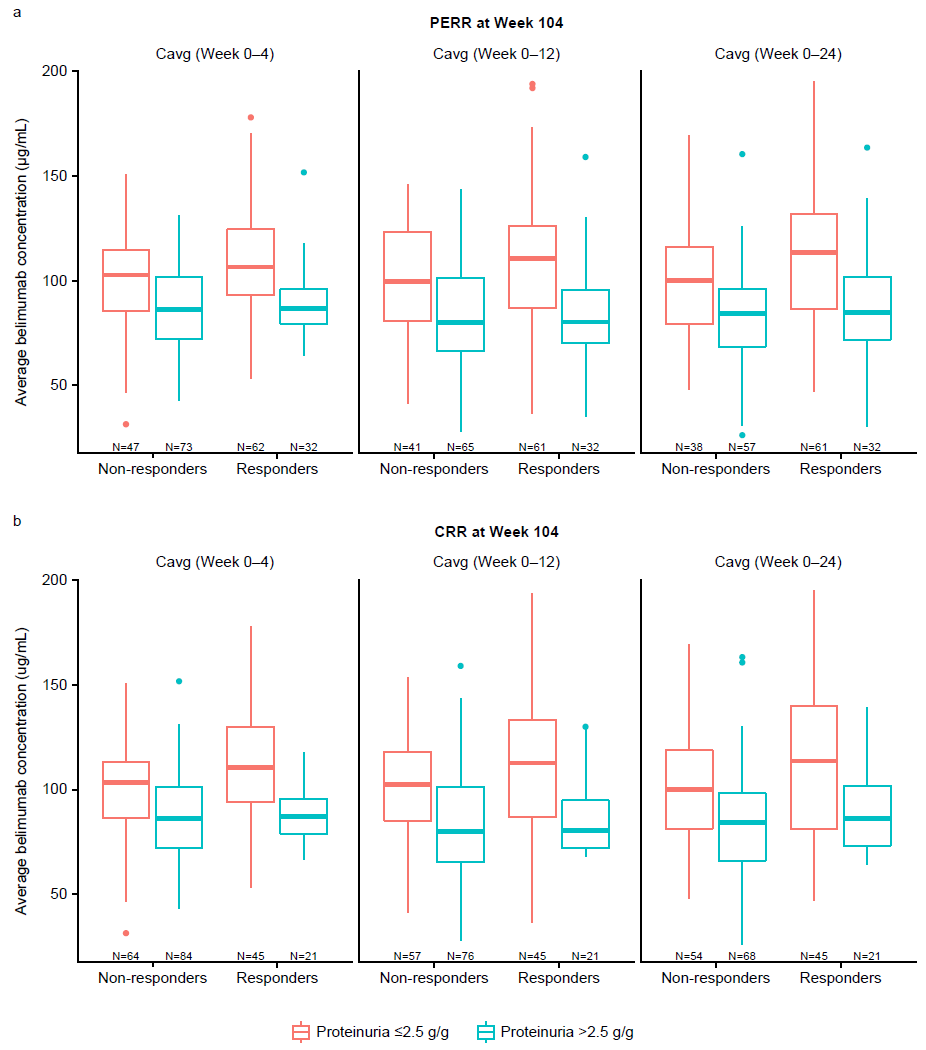


Belimumab Cavg is calculated over Weeks 0 to 4 (left panel), Weeks 0 to 12 (central panel), and Weeks 0 to 24 (right panel). Exposure distributions are shown for the PERR or CRR responder and non-responder patient subgroups, stratified on baseline proteinuria >2.5 g/g (green) and ≤2.5 g/g (red).

Cavg, average concentration; CRR, Complete Renal Response; PERR, Primary Efficacy Renal Response.
